# Planning and Representing Intentional Action

**DOI:** 10.1100/tsw.2003.46

**Published:** 2003-07-01

**Authors:** Bernhard Hommel

**Affiliations:** Department of Psychology, Leiden University, Wassenaarseweg 52, NL-2333 XZ Leiden, The Netherlands

**Keywords:** action planning, intentional action, goal, perception and action, feedback, action effects, action control, will, priming, imitation, mirror neurons, emotion and action

## Abstract

This paper reviews recent approaches to human action planning and the cognitive representation of intentional actions. Evidence suggests that action planning takes place in terms of anticipated features of the intended goal, that is, in terms of action effects. These effects are acquired from early infancy on by registering contingencies between movements and perceptual movement outcomes. Co-occurrence of movements and effects leads to the creation of bidirectional associations between the underlying internal codes, thus establishing distributed perception-action networks subserving both perceiving external events and intentionally producing them. Action plans determine only the general, goal-relevant features of intended actions, while the fine-tuning is left to on-line sensory-motor processing. Action plans emerge from competition for action control between several factors: overlearned habits, perceptual events, and emotional influences, among others. Accordingly, action control represents a balance between personal intentions and wishes on the one hand and environmental affordances and demands on the other.

## PLANNING AN ACTION

Humans perform actions to reach goals, that is, to create or modify some event or state of affairs according to their intentions — otherwise we would talk of movement but not action. Logically, then, intentional, goal-directed action presupposes some sort of (conscious or unconscious) anticipation of the intended goal event, some knowledge about the goal and how it can be achieved. As William James said: "if, in voluntary action properly so-called, the act must be foreseen, it follows that no creature not endowed with divinatory power can perform an act voluntarily for the first time"[1]. Before we can actively aim at a goal, we first need to have experienced that and by what means (i.e., what action) it can be achieved. Once we have acquired this knowledge about movement-effect relationships, we can predict what the consequences of possible actions are and use these anticipations to select a set of movements that most likely produce the effects we aim at. In other words, acquiring movement-effect associations allows us to run internal simulations of possible actions[2,3,4] and base our decisions on how well the outcomes of these simulations fit our current intentions.

An important implication of these considerations is that action planning is basically an off-line activity; planning precedes the action planned. How much it precedes the action may vary considerably. When planning to wake up at a particular time and to go to work the next day, all planning processes are likely to be completed long before the actual action takes place. Accordingly, the action's success does not only depend on the quality of the action plan proper but also on how well the trigger conditions are specified[5,6] and how well the plan is maintained in memory[7]. Creating and executing an action plan can also go hand in hand, especially if the action is not very complicated — just think of reaching for a cup of coffee. In many daily activities, planning and execution may even overlap in time, typical examples being talking — where later syllables of a word or sentence are often planned while the execution of previous ones is underway[8] — or manual action sequences, such as when cooking a meal or grasping an object for further manipulation[9]. In these cases action planning not only involves specifying the goal and the way it is to be achieved, but also needs to take care of a proper sequencing of the action's individual steps and scheduling them in time. A whole number of models have been suggested of how the sequencing part might be solved[10,11,12,13,14,15], and it is not unlikely that different models apply for different types of actions. However, the present review will neglect issues of prospective memory and action sequencing or scheduling and concentrate on the representation and control of simple, single-step actions.

## Evidence of Action Planning

Before addressing how action plans are made up and cognitively represented, we need to be sure that action planning is indeed an important ingredient of human performance. Even though both introspection and logical consideration suggests that actions need to be prepared in advance to some degree, there are alternatives to be considered. Consider the common argument that subjects can be made to carry out almost any task by simply instructing them[16]; does this not show that people can be made to set up a plan or task-set controlling further performance? Not necessarily. For instance, one may imagine that the words making up an instruction automatically activate internal codes of stimuli and responses, and that these then become more and more associated with each other to the degree that performance (first by chance, then by association) obeys the task rules and, hence, leads to reward or the absence of punishment (e.g., through an error beep). If people would acquire these associations very rapidly, their performance would be indistinguishable from true "action planners", even though they would in no way make a plan, i.e., would not anticipate the intended action effect and use this representation to set up a control structure to assemble and steer the action. Fortunately, however, there is converging evidence from at least four lines of research supporting the idea that planning does play an important role in human action control [cf., 17,18].

First, a couple of observations in human patients and experimentally lesioned animals demonstrate that actions can be carried out in the absence of afferent information. People can still move their legs in a goal-directed fashion after the loss of kinesthetic feedback channels even with their eyes closed[19] and draw paintings[20] or synchronize finger movements[21] with most parts of their body being deafferented completely. Likewise, monkeys experimentally deprived from visual and kinesthetic channels can still grasp, run, jump, and climb[22]. Apparently, then, goal-directed action does not rely on external stimuli to trigger or control it, even though most available studies also show that deafferentiation does impair the fine-tuning of actions.

Second, actions consisting of several steps often reveal some sort of anticipation of upcoming elements. For instance, in manual grasping, the size of the to-be-grasped object is commonly reflected in the shape of the hand long before the actual contact takes place[23,24]; to-be-moved objects are grasped in such a way that hand comfort at the end of the move is optimized [9,25,26]; phonemes are spoken differently depending on the phonemes that follow; and errors in speech[27,28] and typewriting [13,29] are often anticipatory in nature, i.e., consist in prematurely producing letters, syllables, and words from the remaining part of the word or sentence.

Third, the time it takes to begin with an action often reflects the action's complexity[30,31,32] or the number of steps it consists of[33,34,35], as well as the number of action features that could not be planned in advance of the reaction stimulus[36,37,38,39]. Thus, the amount of planning required seems to determine when people can begin to carry out an action.

Fourth, reaction times have been found to be sensitive to stimulus information about the expected consequences of an action[40,41,42,43,44,45]. For instance, if keys are pressed to switch on a left and right light, reactions are initiated more quickly if the location of the stimulus corresponds to the location of the light to be switched on[40]. This strongly suggests that intended action goals are considered during action planning already.

Taken altogether, the available evidence provides strong support for the assumption that human actions are preceded by planning processes that take into account the features of the action and/or what effect(s) it is intended to produce.

## Distributed Representation of Action Plans

Spatio-temporal events actions consist of features such as the length and duration of a manual reach, the location the movement it is aimed at, or the pressure exerted on an object grasped. Neuroanatomically, these features seem to be processed in different (sub-) systems of the brain. By using single-cell recordings in the monkey, separable, feature-specific neural systems have been found for the planning of the direction[46,47], force[48], and width[49] of manual movements, and of the planning of manual flexions vs. extensions[50]. In humans, specific electrophysiological markers have been demonstrated for the planning of the hand to be moved[51], and the duration[52] and force[53,54] of manual movements. These observations suggest that action plans are not uniform wholes but are represented in a distributed fashion[55,56,57]. If so, one would expect that different features of an action can be planned independently of each other and at different points in time. Indeed, behavioral studies have revealed that people are able to prepare some features of an upcoming action before knowing others. For instance, human subjects are able to reduce the time to plan a manual movement if they are precued about the hand to be used, and the direction or width of the movement[36,37,38,39]. Likewise, action planning can be facilitated by priming particular action features[58,59], as discussed below in more detail. Thus, action plans are likely to consist of networks of codes that represent the features of the planned action[57,60].

## Action Planning in Terms of Perceptual Effects

Considering that action plans can be assumed to consist of codes representing their features, two important questions pose themselves: How, then, do people activate those codes in the process of planning an action and how does activating a feature code translate into real action? These questions have a long, respectable tradition. It was Lotze[61] who first approached them in a systematic fashion. Based on his own introspections, he concluded that humans are basically ignorant with respect to their motor capabilities and, indeed, if asked exactly how we tie our shoes, ride a bike, or produce a vocal sentence, we commonly do not have much interesting to say. But still, we can make our muscles do the things we want them to do — how is that?

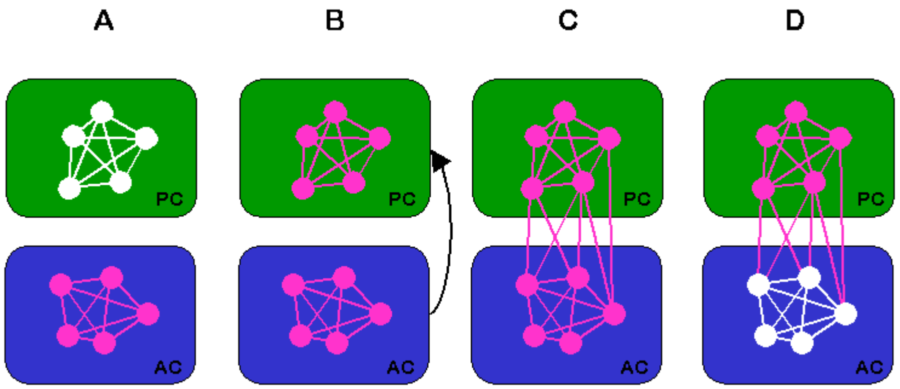

**FIGURE 1.** A two-stage model of the acquisition of action control. Stage 1 is comprised of phases A–C. (A) Random activation of some pattern of action codes (AC) produces overt movement. (B) The reafferent effects of the movement are perceptually coded by activating a pattern of perceptual codes (PC). (C) Repeated co-occurrence of AC and PC leads to the creation of an associative, perception-action network. At Stage 2 (phase D) AC can be intentionally accessed by activating the associated PC, i.e., by anticipating the intended action effects.

Lotze's solution, later picked up by James[1] and now known as the *ideomotor principle*[7,62,63], consists of a two-phase process (see Fig. 1). First (i.e., as a baby or a novice of a given task), we move in a more or less random fashion, that is, without being able to predict or aim at particular action effects. However, moving always produces particular sensory effects that we can perceive. The codes of these perceived effects are automatically associated with the motor patterns producing them, thereby creating bidirectional movement-effect associations[42,64,65]. Once acquired, these associations can be used either way, which allows us to enter the second phase: Now we can "imagine" the effects we wish to achieve (i.e., our current action goal) and this is sufficient to activate the associated action. In other words, we can use anticipations of the sensory effects a particular movement has been learned to produce to set up the plan to carry it out again to now bring about these effects intentionally.

One implication of this ideomotor approach is that imagining an action should lead to processes also involved in action planning. Indeed, studies on motor imagery suggest that this is the case[66]. In these studies, people are commonly asked to imagine performing a particular action, often while being brain-scanned. When they do so, they are likely to remember how it feels like to carry out the respective movements, hence, people are likely to activate the sensory representations the imagined movement would (and actually did) bring about. Accordingly, it may not be too surprising that the duration and time course of imagined movements corresponds closely to those of real movements [e.g., 67,68,69]. However, imagining the performance of an action can be shown to activate brain structures involved in action planning, especially the supplementary motor area (SMA)[70,71].

Further evidence comes from studies on action perception. According to the ideomotor principle, action planning involves activating codes that originated from self-perception on previous occasions, that is, codes representing the perceived effects of one's own actions. Perceiving oneself and perceiving another person to perform an action is certainly different, especially because tactile and kinesthetic impressions will be lacking in the latter case. Nevertheless, the two events do share many features, so that following the ideomotor principle, their cognitive/neural representations should overlap to a large degree. As a consequence, action planning should be facilitated if the stimulus calling for an action shares features with that action. Indeed, it is long known that feature overlap between stimuli and responses speeds up reaction time, the so-called *stimulus-response compatibility* effect[72,73]. For instance, choosing between a left and right response is easier if the corresponding stimulus also appears on the left or right side, respectively[74,75]; moving a finger up or down goes faster in view of a up- or down-going finger[76]; flexing and extending one's hand is initiated more quickly vis-à-vis a picture showing a flexed or extended hand[77]; and uttering a word is easier if signalled by that word[78,79]. That is, if one can already see or hear what needs to be done, planning to do it is facilitated, apparently because perceiving an event that is similar to the required action already activates part of the codes that make up the appropriate plan[10,18,41,60].

Support for the at least partial overlap of perceptual and action-related codes comes from single-cell and brain-imaging studies. Rizzolatti and colleagues have identified areas in the inferior premotor cortex of the macaque monkey where cell activity codes particular actions independently of whether these are observed or self-performed by the monkey[80]. These so-called *mirror neurons* are sensitive to object-oriented actions, such as grasping a piece of food, but more or less silent to presentations of either the object or the action alone[81]. The human homologue of this brain area seems to host cells with comparable characteristics. Brain-imaging studies show activity in this area while human subjects either perform or watch object-oriented actions[82], while no activity is registered when subjects see meaningless movements[83]. If action-related activity does occur, it is rather specific in being restricted to those areas that would be involved if the observer carried out the watched action him- or herself[84].

Taken together, these behavioral and neurophysiological findings provide strong support for the old ideomotor principle, hence, for the assumption that action planning takes place in terms of — i.e., by making use of the codes of — their perceptual effects.

## Acquisition of Action-Effect Associations

As mentioned above it was William James who pointed out that "no creature not endowed with divinatory power can perform an act voluntarily for the first time"[1]. The reason is that intentional action presupposes a goal and, hence, some expectation about the effect of the action, almost by definition. Expectations, however, presuppose knowledge about what to expect, which again requires some degree of previous experience regarding what types of activity produce what kinds of effects. As there is evidence of at least rudimentary forms of intentional action from the very first weeks of life on[85,86], the learning mechanisms underlying the acquisition of action-effect associations must be working quite efficiently at this time already. Indeed, learning of means-ends relations has been demonstrated in babies as young as 5 weeks of age. For instance, babies are able to systematically adapt their sucking behavior (i.e., the pressure applied to a pacifier) to increase the optical clarity of a movie presented to them[87] or to modify the pitch of a tone[88]. Slightly older, 2-month-olds, have been shown to control the motion of a mobile hanging above their crib by exerting a particular amount of pressure on their pillow[89] or by moving their legs accordingly[90]. These findings suggest that experiencing a novel, movement-contingent effect leads to the acquisition of movement-effect associations, just as claimed by the ideomotor approach to intentional action.

As further claimed by the ideomotor approach, movement-effect associations are apparently bidirectional. For instance, once a baby has learned how to influence a mobile by moving his or her leg, presenting a moving mobile at some later occasion also makes the baby move the leg again[91,92]. Similar effects have been observed in adults. For example, if adults experience repeatedly that a left and a right keypress is followed by a low- and high-pitched tone, respectively, presenting these tones as irrelevant primes in a later, independent task facilitates performing the corresponding keypress[41,93]. Even the outcome of response decisions can be primed by action effects. Assume, as in the previous example, people have learned that a left keypress produces a low, and a right keypress a high tone. If they then are free to perform one or the other keypress in response to randomly presented low and high tones, they are more likely to choose that keypress that previously produced this tone[42]. That this is not just a high-level decision effect has been demonstrated in a recent PET study. After having acquired action-contingent tone effects adults were asked to merely listen to those and other tones while being brain-scanned. Only two brain areas showed activity that correlated with the frequency of action-effect tones: the SMA and the right hippocampus[94]. Thus, activating the codes of acquired action effects has a direct impact on brain structures responsible for action planning.

## PERFORMING AN ACTION

Action planning takes place before an action begins and it therefore relies on available knowledge about past action-effect relations to predict the best means to produce the intended effect in a given situation. But predictions may fail and they are likely to do so the less experienced the acting individual is and the more dynamic the situational circumstances are. Moreover, not all parameters of an action are relevant for its success: If one intends to take a sip of juice it does not matter exactly how the glass is grasped and moved towards the mouth — if one only gets that drink. These considerations suggest that action plans are incomplete in specifying only what is important for reaching the intended goal and what can reliably be predicted before the limbs begin to move. Indeed, several authors have argued on both theoretical and empirical grounds that action planning underspecifies the intended action and leaves open "slots" to be filled by on-line sensory information[24,95,96,97].

Strong support for the idea that off-line planning and on-line specification may interact in bringing about goal-directed actions is provided by studies using *double-step* stimuli[98,99]. In such studies, subjects are asked to move one of their (unseen) hands to a light target. In some trials, the target is moved for some centimeters without notice. As this is arranged to happen during an eye movement of the subject, subjects are unable to see that movement and, indeed, commonly report that the target did not move at all. Nevertheless, the hand adapts to the new situation immediately and starts moving towards the new target location without measurable hesitation[99,100]. Obviously, the hand "knows" more than the subject is able to report and, hence, more than the action plan entailed. Also, if we assume that action planning costs time (as commonly expressed in reaction time measures), the observation of zero time costs to update the target location strongly suggests that (precise) target location was not part of the off-line plan but was specified on-line.

Based on these and many other empirical observations Milner and Goodale[101] have proposed a neurocognitive model of visuomotor manual action, in which off- and on-line channels of information processing are considered. In their model, on-line sensory-motor processing proceeds along the dorsal visuomotor pathway that begins to segregate in the visual areas V1 and V2 and then connects to the motor cortex via relay stations in the posterior parietal cortex. This channel is assumed to deliver information about grasp- and reaching-relevant visual information, such as location and size, which directly feeds into systems responsible for hand control. Milner and Goodale claim that information processed along this route is not available to consciousness, which apart from the double-step studies mentioned above fits with the observation that patients suffering from form agnosia are able to properly grasp objects they, at the same time, are unable to identify[102]. The other, off-line channel, is assumed to run from V1, V2, V3, and V4 straight to the inferotemporal cortex. It has access to memory, is accessible by consciousness, and its main function is proposed to be restricted to perception. If this channel is impaired, as in optic ataxia, people may be able to identify an object but at the same time be unable to grasp it properly[103].

Milner and Goodale's two-pathway model has been widely discussed and in some cases challenged[104,105,106,107,108]. For instance, the authors assume that manual grasping is not affected by visual illusions (as the former is processed dorsally, the latter ventrally), but such effects do occur under some circumstances[107]. Moreover, the model has not much to say about how the two streams interact to produce coherent action [but see 109], and it seems to underestimate the degree to which they interact[110]. Action planning is ignored altogether[111], so that the model seems to attribute most of the action control to the stimulus. However, taken more generally, Milner and Goodale's distinction between a memoryless on-line channel that provides on-going actions with

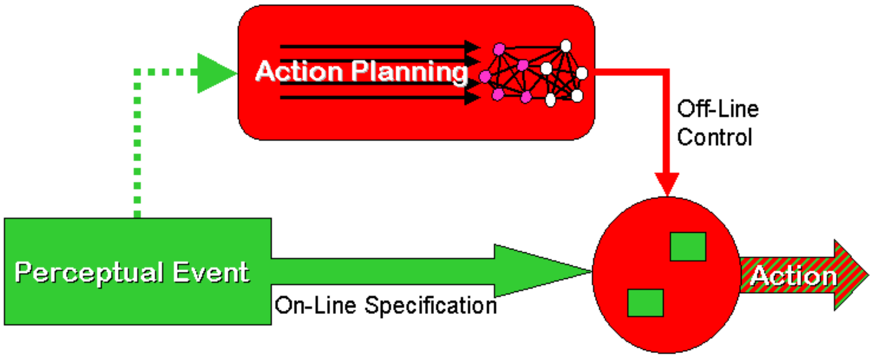

**FIGURE 2.** The interaction of sensorimotor processing and action planning. Action plans are worked out off-line and then, after completion, specified by on-line sensorimotor processing.

the most up-to-date sensory information and a memory-based off-line channel that is more sensitive to the thoughts and intentions of the individual is in good agreement with, and provides a useful summary of, the data available so far[111,112]. This leaves us with a picture along the lines of Fig. 2, which sketches the distributed functions of an off-line perception-action pathway that sets up general plans for goal-directed action, and an on-line sensory-motor channel that fills in the slots left open by the action plan.

## COMPETITION FOR ACTION CONTROL

The available evidence suggests that action plans are internally generated, underspecified, and defined in terms of codes of the features of intended action effects. Ideally, an individual might have thought of considering some action goal, retrieving from memory how the goal had been achieved in the past, selecting the means that seem most appropriate under present circumstances, implementing the plan, and having on-line sensory information fill in the parameters not yet specified. But this does not reflect our daily experience. We commonly have more than one goal, often carry out several actions at once, sometimes have difficulties in deciding how to reach a goal or, if we have decided, forget what it was, and so forth. Hence, there is an on-going competition for action control between a whole multitude of action tendencies[113]. Even planning a single action can be affected by this competition, be it quantitatively (e.g., by lengthening or delaying the planning process) or qualitatively (e.g., by triggering an inappropriate action). One reason for why our brain is so sensitive to internal competition is that it faces a basic dilemma[114]: While it is reasonable to follow our past experience and to shield an action plan against interference, we cannot afford to be immune to new information, situational constraints, and other, perhaps more interesting action opportunities. Hence, it makes good evolutionary sense to keep some balance between defending present goals and action plans against interference and staying open to new information all the time. The following three are likely to be the most relevant factors affecting this balance.

### Will and Habit

One of the first researchers approaching issues of intentional action control experimentally was Narziss Ach[115,116]. Analyzing the will at work, so he reasoned, requires setting it in opposition to previously acquired habits that need to be overcome. This way it may be even possible to measure willpower: The easier will would overcome an opposing habit the stronger it must be, the more so the more overlearned the habit. Along these lines, Ach had subjects acquire particular habits, such as learning to produce rhymes to nonsense syllables, to different degrees (i.e., for varying days of practice). Then subjects were asked to perform a different type of action, such as reversing the order of the letters, to the same or to new stimuli. The measure of interest was whether reaction times would differ between these conditions, that is, whether people would have a harder time performing a response to a stimulus if that stimulus had been given a different response earlier. Indeed, this is what Ach found: Responding to a stimulus was more difficult (in terms of reaction times and errors) if it was previously paired with a different, now incompatible response[116]. Follow-up studies replicated and extended these findings[117,118,119] but as none was translated into English they fell into oblivion[120]. However, the basic idea that intentional processes and habits are (often opposing) factors that compete for action control has survived and now forms a major ingredient of theories of automaticity, stimulus-response compatibility, and action control.

Automaticity theories often distinguish between a slow, intention-driven, capacity-demanding algorithmic route from stimulus to response on the one side and a fast, memory-driven, capacity-free, associative route on the other[121,122,123,124]. Tasks are thought to become automatized by acquiring stronger and more reliable stimulus-response associations[122], or a greater number of episodic stimulus-response memory traces[123], so that the algorithmic route is less and less needed and stimulus-response translation becomes faster and more efficient. However, the stronger stimulus-response associations get more serious as they compete with intentional processes for action control in case the currently intended response to a stimulus is not the most practiced one, very much along the lines of Ach's original reasoning.

The idea that practice makes the practiced task more autonomous, and that this may interfere with less practiced tasks, has also been applied to stimulus-response compatibility effects. For instance, several authors have attributed the Stroop effect (interference of color-naming responses by incongruent color names) to competition between the intended but less practiced color-naming response and the inappropriate but well practiced color-word-reading response[125,126,127]. In agreement with this approach, varying the relative strength of incompatible stimulus-response associations systematically allows one to predict the direction and relative size of Stroop-type effects[128]. Relative-strength accounts have also been suggested for spatial stimulus-response effects, such as the spatial stimulus-response compatibility effect proper (where stimulus location is nominally relevant) and the Simon effect (where stimulus location is nominally irrelevant)[129,130,131]. Thus, people may find it easier to respond to left and right stimuli with a left and right response, respectively, because the spatial codes of corresponding stimuli and responses are associated more strongly. Again, this reasoning finds support from studies where compatibility effects were assessed after having subjects practice spatially compatible or incompatible mappings[132,133].

A third area where competition between intentional processes and acquired habits plays a major theoretical role deals with the implementation of, and the switch between, task sets. It is a common observation that people find it difficult to switch rapidly from one task to another, such as from reading the word of a Stroop-type color-word combination to naming its color, and vice versa[134,135]. According to the influential model of Shallice et al.[136,137], this is because stimuli trigger their overlearned response. This works well if the trigger is indeed the intended response but requires action from a (not further explained) *Supervisory Attentional System* if another response is needed — hence, again, will and habit in competition for action control. Later models attempted to be more explicit with regard to the will component, which is now seen to be strongly connected to working memory[138,139] and the representation of task context[140,141] The basic idea underlying these models is that people are able to acquire context- or task-specific stimulus-response associations, or at least the means to modulate stimulus-response associations in a context- or task-specific fashion, so they can tailor their task set to the situational requirements. In combination with appropriate reward systems that strengthen task-specific processing in case of success and/or favor competing processes in case of failure[142,143], such modeling attempts are likely to enable us to decompose the homunculoid concept of *will* and replace it with less fancy processing models. The main lesson remains, however: Intentional action planning is not immune to opposing action habits and may sometimes even loose the competition for action control, the more likely the more practiced competing action tendencies as compared to the intended action.

## Perceptual Priming

Action planning is an off-line process by definition. Indeed, it is the major advantage of action planning that it allows the effective decoupling of stimulus input and action output, thereby allowing for truly anticipatory, goal-directed action instead of mere reaction to the present stimulus conditions. Accordingly, action planning will often rely more on internal trigger conditions, such as intentions, wishes, and motives, than on external triggers — even though the completed plan may well define external conditions to trigger its eventual execution[5,6]. But this does not mean that external conditions have no impact on the planning process and its ultimate outcome. Indeed, most of our current insights about action plans come from studies in which such external influences were investigated, especially the impact of stimulus-response compatibility and of stimulus-induced imitation.

As pointed out above, stimuli tend to facilitate actions with which they feature overlap: left and right stimuli facilitate left- and right-hand manual actions, static and dynamic gestures facilitate movements that look the same, and words faciliate naming them[74,75,76,77,78,79]. Similarly, watching an object-oriented action activates motor structures in the brain that are involved in producing those actions[80,81,82,83,84]. The impact of feature overlap is specific to the goals and meanings of actions, not (just) motor parameters. Hence, even the feature-overlap between a stimulus and an effect a given action has been learned to produce speeds up planning and executing that action[40,41,42,43]. Thus, what matters is a feature overlap between how a stimulus and an intended action are cognitively coded, which strongly points to action planning as the locus of the impact (the upper route in Fig. 2). Action planning seems to involve activating codes that perceiving an overlapping stimulus delivers already.

On the one hand, this may be seen as a mere, rather unfortunate side-effect of the way perceptual events and action plans are coded. On the other hand, however, several researchers have pointed out that facilitation by feature overlap between perception and action planning also supports imitative learning[63,82,83,144,145], which especially in early development is a function of obvious and widespread use. This does not mean that imitation of perceived action is easy or overly direct. Indeed, a whole bunch of transformations need to be applied before a visually perceived action can be carried out. Assume, for instance, one attempts to learn skiing from watching an expert. To transform what is seen into what needs to be done requires relating visual codes to motor, or at least kinesthetic and tactile codes; transforming the allocentric frame of reference in which the perceived event is presumably coded into the egocentric reference frame that likely underlies action planning; transforming the body height and bone lengths of the model into one's own body measures; taking care of the differences in ski length and material; and so forth. It is not very likely that all the information needed to carry out all these transformations and adaptations can be extracted from the visual input, so that the action plan will be underspecified to a considerable degree. As this argument is likely to apply to many other examples of imitative learning, we need to assume that imitation operates on a relatively abstract level.

Indeed, recent research reveals that infants and children imitate what they perceive as the action's actual goal but not how this goal is reached in the particular situation[146,147,148]. For instance, if kids had watched an adult pressing a switch with her nose they will also tend to use their own nose to press the switch[149]. However, this is the case only if there is no indication that this particular way to reach the goal might have been accidental: If the adult is visibly prevented from using her hands while pressing the key, kids will use their hands to operate the switch. Hence, children do not imitate movements but goals[145]. Interestingly, the same seems to be true for monkeys and apes, at least if reared in the wild[150,151]. That is, the imitation capabilities of the only species in which mirror neurons have been demonstrated so far are restricted to action goals. This might suggest that the main function these neurons have is to code action plans at a level of abstraction that is appropriate for specifying the goal-relevant features but leaves the fine-tuning to direct sensory-motor loops (see Fig. 2).

## Emotional Priming

Many actions we perform serve their underlying goal in obvious ways: we reach for a glass to quench our thirst, speak and listen to communicate with someone, and walk or ride to get to another place. Some decisions to reach a goal in one particular way are suggested by efficiency, others are dictated by habit, and yet others result from environmental constraints. But there are also decisions that have less obvious reasons, decisions that are made because they, or the action they imply, "feel good".

Damasio and colleagues have argued that the mechanism underlying such "emotional" aspects of action planning operate on *somatic markers*[152,153]. The idea is that representations of actions entail, among other things, markers of how it commonly feels like performing that action. When considering what kind of action to select in a particular situation, the individual can check (i.e., activate) these markers to predict whether he or she will feel good or bad when performing the corresponding action. Although this is unlikely to be a major criterion in many types of tasks and situations, it may help to make a reasonably good decision in cases of uncertainty. Indeed, the logic underlying the use of somatic markers is not too different from that of action-effect codes as discussed above: the latter represent the perceptual effects, the former the emotional effects of an action[154].

There are several indications that somatic markers can play a role in action planning under uncertainty. To tap into these decision processes, people are commonly confronted with a card-drawing game, in which complicated contingencies exist between different card decks and financial gains and losses. Healthy subjects learn such contingencies rather quickly and, thus, are able to increase their "game budget"[155]. After some learning they show increases in galvanic skin conductance responses (GSCRs) whenever they are about to draw from a disadvantageous deck, suggesting some (apparently sometimes subthreshold) somatic source discriminating good from bad choices. In contrast, patients suffering from lesions in the ventromedial part of the frontal cortex perform particularly badly, they often loose their game budget and take considerable "loans". They also fail to show any difference in GSCRs when drawing from advantageous and disadvantageous decks[156]. Thus, an intact frontal lobe seems to enable people to anticipate the emotional consequences of possible actions. Further evidence that emotional action effects play a role in action planning comes from studies showing that actions are initiated more quickly if the (positive or negative) valence of their acquired effects (e.g., electric shocks, or smileys and grumpys) corresponds to the valence of the stimulus triggering the action[157].

Taken altogether, the available evidence suggests that action plans emerge from competition of a whole number of action tendencies and sometimes represent a kind of compromise between them. These tendencies may arise from several sources: internal sources, like goals, habits, and anticipated perceptual or emotional action effects, and external sources, such as observed actions or action-related events. Thus, action control represents a balance between personal aims and wishes on the one hand and external affordances and demands on the other.

## BIOSKETCH

**Bernhard Hommel**, Full professor, Chair of General Psychology, is interested in all sorts of interactions between perceptual processes and action control, on both the emergence of cognition from such perception-action interactions and their cognitive control. Related interests concern the dynamics of information processing and the integration of distributed information.

Other research interests include: perception and action, emotion and action, representation of action plans, acquisition and development of intentional action, multiple-task performance and executive control, integration of distributed information, attentional control and control of attention, development of cognitive functions across lifespan, and spatial cognition.
